# Ecological functional zoning for mineral resource development in Xizang, China

**DOI:** 10.1371/journal.pone.0322418

**Published:** 2025-05-09

**Authors:** Xiangying Jia, Chonghao Liu, Jing Liu, Jianan Zhao, Xiu Wang, Niannian Li, Tianjiao Li

**Affiliations:** 1 Institute of Mineral Resources, Chinese Academy of Geological Sciences, Beijing, China; 2 Research Center for Strategy of Global Mineral Resources, Chinese Academy of Geological Science, Beijing, China; 3 Guangdong Mineral Resources Exploration Institute, Guangdong Geological Bureau, Guangzhou, China; Government Degree College Totakan, PAKISTAN

## Abstract

Xizang is an important base of mineral resources, hosting abundant deposits. However, its unique and fragile ecological environment has long constrained resource development and utilization. Achieving a balance between ecological protection and mineral resource exploitation has become a pressing issue. This study proposes a novel approach for ecological functional zoning to provide scientific evidence for mineral resource development in Xizang. The study employs remote sensing image interpretation and single-factor ecological indicators analysis to construct a comprehensive ecological environment assessment system, scientifically delineating ecological function zoning for mineral resource development in Xizang. The results indicate that Xizang can be divided into five functional zones: (I) Northern Plateau Desert Zone, (II) Plateau Grassland Zone, (III) Plateau Mountain Zone, (IV) High Mountain-Forest Zone, and (V) Eastern Canyon Zone. Zone I and Zone III exhibit relatively favorable conditions for development but also face significant ecological vulnerability. Therefore, development activities in these areas must be rigorously controlled to minimize environmental disturbances. Zone II and Zone IV impose substantial constraints on resource development due to the heightened sensitivity of their ecosystems, necessitating stringent conservation measures. While Zone V demonstrates strong ecological restoration capabilities, it remains highly susceptible to water resource contamination and soil erosion risks. The innovative outcomes of this study lie in integrating comprehensive regional zoning and quantitative ecological environment assessments, providing an actionable framework for the coordinated development of mineral resources and ecological protection, thus advancing the scientific and refined management of resources and the environment.

## 1. Introduction

In the context of a globalized economy, the strategic importance of mineral resources is increasingly significant, often referred to as the “food of industry” and “cornerstone of modern construction” [1]. With the advancement of science and technology and the expansion of industrial activities, the demand for mineral resources has risen sharply, making their exploration and development a focus of international attention [2]. As one of the world’s largest consumers of mineral resources, China faces a substantial supply-demand gap. Despite its vast territory and abundant mineral resources, China’s rapid economic development has led to increasing dependence on overseas mineral resources, posing potential risks to resource security and sustainable development 3. Against this backdrop, Xizang in southwest China has emerged as a vital mineral resource base due to its rich deposits of copper, chromium, lead, zinc, gold, and silver [4,5]. However, given Xizang’s high-altitude plateau setting and its inherently fragile ecological environment, achieving a sustainable balance between mineral resource exploitation and ecological conservation demands a heightened emphasis on environmental protection measures [6,7].

Most previous studies have focused on macro-level environmental impact assessments, yet they have largely overlooked the detailed analysis of how specific ecological constraints influence the exploitation and development of mineral resources [8–10]. This gap is particularly critical when it comes to functional zoning for mineral resource exploitation, as it directly affects the evaluation of the suitability of mineral resource development. However, these essential factors have not been thoroughly examined in the context of Xizang. This shortcoming significantly restricts the ability to conduct a comprehensive scientific assessment of the ecological suitability for mineral resource exploitation. Xizang is normally regarded as a homogeneous region, neglecting the diverse ecological environment and mineral resource characteristics of different areas. This lack of regionally differentiated study restricts the ability to develop targeted strategies that balance ecological and mineral resource development [11,12]. There are significant differences in ecological sensitivity and resource distribution across various areas of Xizang, which requires development strategies tailored to local conditions. Moreover, many studies have primarily focused on the negative impacts of development activities on the environment but lack a systematic approach to ecological priority decision-making [13,14]. This deficiency makes it challenging to balance the needs of ecological protection and resource development in actual practice. Therefore, a scientific approach that can comprehensively consider ecological constraints and the feasibility of resource development is needed to achieve sustainable resource development.

To fill the gap, the Analytical Hierarchy Process (AHP) and spatial analysis methods were employed to construct a comprehensive ecological environment assessment system by integrating remote sensing image interpretation and single-factor eco-environmental index analysis [15]. This system can systematically assess the ecological constraints faced by the development of mineral resources in different areas of Xizang and provide detailed ecological background information for mineral resource development. Although the AHP method is relatively simple, its advantage is particularly evident in this special region of Xizang. AHP can decompose complex decision-making problems into multiple levels and criteria, performing qualitative and quantitative analysis through expert experience [16]. In macro-level planning, the AHP method can more accurately reflect the actual situation by decomposing complex problems into a multi-level structure and integrating expert experience and qualitative analysis. In particular, in scenarios involving comprehensive assessments of multiple factors, AHP can effectively balance the weights of different factors, thereby providing more scientific and practical support for decision-making [17]. This method is particularly suitable for factors that are difficult to quantify, such as environmental impact and social acceptance, thereby making the decision-making process more comprehensive and detailed [18]. The advantage of AHP lies in its reliance not on complex mathematical models but on a structured decision-making framework, which gives the method a high degree of flexibility and adaptability. Decision-makers can adjust the hierarchical model according to actual conditions to fit different decision-making environments and needs. The ecological environment in Xizang is complex and fragile, and a variety of ecological constraints need to be considered. For example, in this study, by conducting a hierarchical analysis of topographical factors (such as elevation, slope, and freeze-thaw erosion) and ecological factors (such as precipitation, vegetation coverage, and land use types), and assigning corresponding weights to each factor, AHP can comprehensively consider the actual impacts of these factors on mineral development. This method allows experts to assign weights to these factors based on their extensive experience and expertise. This experience-based expert weighting approach is particularly advantageous in the context of Xizang, where the ecological environment is highly complex and data availability may be limited. Compared to idealized weighting calculations, which may not fully capture the nuances of the local ecological conditions, the experience-based weighting by experts provides a more reliable and contextually appropriate assessment. By integrating the opinions of multiple experts, AHP can effectively reduce the bias of individual decision-makers, thereby enhancing the objectivity and fairness of decisions. The method’s transparency and consistency check mechanisms further ensure the rationality and reliability of the decision-making process [19]. It not only simplifies the evaluation process but also, based on expert experience and real data, more accurately reflects the ecological vulnerability and resource development potential of different regions [20]. This study can better balance the needs of ecological protection and mineral resource development, thus providing strong support for the sustainable development of Xizang.

Unlike previous studies that mainly focused on the impact of development on the environment, this study proposes a scientific decision-making method of ecological priority by systematically analyzing ecological constraints. This method fully considers ecological risks when assessing the suitability of mineral resource development to ensure that resource development activities are conducted under the premise of ecological protection [21]. This balanced approach provides a scientific basis for policymakers and industry practitioners to develop more rational and sustainable resource development strategies, complementing the shortcomings of existing research in a systematic eco-priority decision-making approach. Combining the ecological environment indicators with the existing metallogenic belts and the distribution of important mineral resources in Xizang, this study proposes an ecological functional zoning for mineral resource development. This zoning approach ensures that development activities can be adapted to the specific ecological context of different regions, promoting sustainable and balanced development. This study identifies certain areas where mining can be carried out with minimal ecological impact while also highlighting areas that need to be strictly protected. This contribution compensates for the lack of regional differentiation in previous studies.

The purpose of this study is to systematically analyze the specific constraints of the ecological environment on the development of mineral resources and propose ecological functional zoning to realize the coordinated development of mineral resource exploitation and ecological conservation in Xizang. These innovative contributions make up for the shortcomings of existing studies in the analysis of ecological constraints, regional differentiation, and systematic ecological priority decision-making methods. By emphasizing ecological priority and scientific planning, this study offers new ideas and methods for the coordinated development of resource exploitation and ecological protection, providing an important reference for policymakers and industry practitioners. This study fills the gap in existing research by constructing a comprehensive ecological environment assessment system, proposing a regional functional zoning scheme for mineral resource development, and proposing a scientific decision-making method for ecological priority, providing scientific guidance for the development of mineral resources in Xizang.

## 2. Materials and methods

### 2.1. Study area

Xizang Province is situated within the core area of the Qinghai-Xizang Plateau in southwestern China. Xizang exhibits diverse geomorphological features, including mountain ranges, basins, plateaus, and deeply incised valleys. Influenced by natural factors including high altitude, low temperatures, arid climate, oxygen scarcity, and persistent winds, Xizang supports distinctive and vulnerable alpine ecosystems ranging from cold deserts, alpine meadows, alpine steppes, alpine shrubs, to montane forests [22]. The region is rich in water resources and is divided into the eastern outflow and central-northern inflow regions. The latter predominantly features lakes and wetlands, with the second-largest wetland area in China. Forested areas are predominantly located in the southeast, harboring extensive primary forests. Arable land is predominantly situated in the valleys encircling Lhasa, which have favorable hydrothermal conditions. Xizang also possesses vast glacier resources, acting as a vital hydrological reservoir for China and Asia [23,24]. However, in the context of global warming, the accelerated retreat of glaciers in Xizang poses a significant risk to the water security of China and Asia [25–27]. Xizang’s ecological environment is experiencing varying levels of degradation, a situation that demands immediate attention and action. The evidence is unequivocal: glacial melting and heightened river discharge, diminishing lake and wetland areas, deteriorating grasslands, intense soil erosion, aggravated soil desertification, endangering biodiversity, coupled with an uptick in geological calamities including landslides, debris flows, and collapses [28,29]. In summary, Xizang’s ecosystem, intricate and distinctive, confronts escalating threats stemming from inherent environmental limitations and escalating anthropogenic pressures.

**Fig 1:**
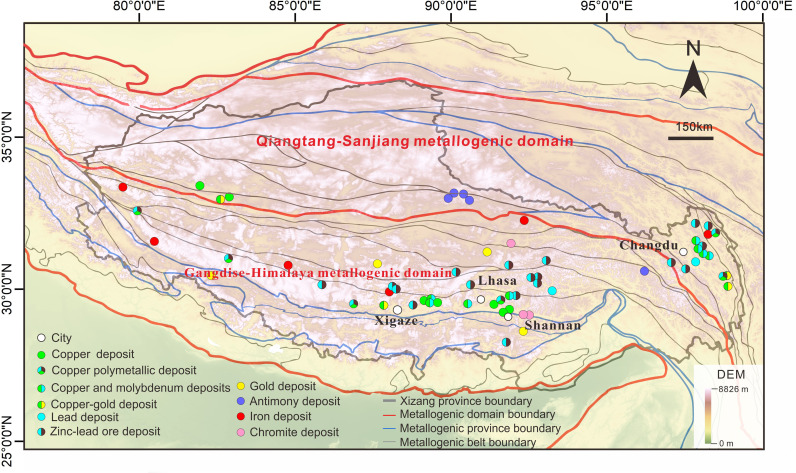
Image of major mineralization belts and important large-scale ore deposit distributions in the Xizang region. The image was created with ArcGIS 10.2, URL: http://www.esri.com/software/arcgis/arcgis-fordesktop. DEM data come from National Tibetan Plateau/ Third Pole Environment Data Center [33]. Geologic structure sketch is modified from ref [34,35].

Geologically, Xizang forms the main body of the eastern segment of the Tethyan metallogenic domain. Its geological history includes the evolution of multiple island arc-basin systems from the Paleozoic to the Mesozoic and intracontinental convergence and orogenic processes in the Cenozoic [30]. The unique tectonic setting provides excellent metallogenic geological conditions. Xizang has large-scale, recently formed, diverse, and well-preserved metallogenic characteristics, making it one of China’s most resource-concentrated regions. The region contains several metallogenic belts, including Nyainqentanglha, Bangong Co-Nujiang, Gangdese, Himalayas, and Sanjiang [4,31,32]. Over 3,000 metallic deposits have been discovered in this area, encompassing 102 types of minerals. These metals include copper, lead, zinc, chromium, gold, and silver, with Xizang’s chromium resources accounting for 66.6% of the national total, copper resources constituting 30.5%, and the proven gold resource reserve exceeding 1,000 t. The significant mineral resources in Xizang are primarily located in the Lhasa-Shigatse-Shannan area and the Changdu region of eastern Xizang, with copper, chromium, lead-zinc, and lithium being the main development targets. Although the development primarily involves extraction and raw ore processing, the mineral processing industry chain remains incomplete (**Fig 1**).

Xizang’s mineral resources are pivotal to the nation’s economic and social development. The annual copper mining scale in Xizang reaches 17.8 Mt [4]. The essential copper deposits include the Yulong copper deposit, Qulong copper deposit, and Jiama copper-polymetallic deposit [36]. The annual lead-zinc mining scale is over 4.35 Mt (Xizang Geological and Mineral Exploration and Development Bureau). Among the deposits, the Yaguila lead-zinc deposit and Bangzhong zinc-copper deposit are the largest. The Zabuye saline lake deposit is the first lithium base in China, with an annual mining scale of 117,000 t and an annual production capacity of 5,000 t [37]. The annual molybdenum mining scale is over 420,000 t [38]. Antimony deposits are primarily distributed in the Qiangtang-Sanjiang and Himalayan metallogenic belts, recognized for their rich mineral endowments. Although the Gangdese area currently hosts smaller deposits, they possess considerable untapped potential. The proliferation of mining enterprises and the advancement of mining projects carry profound implications for the nation’s economic and social fabric. They offer essential underpinnings to China’s capacity to navigate the intricate and fluctuating dynamics of the international arena.

### 2.2. Indicators and methods

Previous studies on the ecological zoning of specific regions have extensively employed key ecological impact factors, such as the Digital Elevation Model (DEM), slope, Normalized Difference Vegetation Index (NDVI), annual average precipitation (Precipitation), and land use types as the foundation for analysis [39–42]. These studies have unveiled human activities’ profound impact on regional ecological environments and provided a solid scientific underpinning for regional ecological space management. In this study, we seek to evaluate the ecological environment in Xizang thoroughly, building upon previous research. Considering Xizang’s unique geography and climate, this study continues to use traditional ecological factors (i.e., elevation, slope, NDVI, rainfall, and land use types) while introducing the factor of freeze-thaw erosion to more accurately reflect the ecological conditions and environmental changes in the area. In light of the ecological damage caused by mineral resource extraction, this study integrates geological structural units and the above-mentioned factors to conduct a univariate ecological environment evaluation based on topographic and ecological variables for the functional zones of mineral resources in Xizang (**Table 1**). This study aims to provide a comprehensive environmental impact assessment for resource development in the region, thereby achieving a balance between maintaining ecological integrity and the sustainable use of resources.

**Table 1:** Criteria and data sources within the ecological environment indicator system.

Data	Discrimination Criteria				Data Source	Spatial resolution
	Level 1	Level 2	Level 3	Level 4		
Elevation (m)	≤1000	(1000, 2000)	(2000, 5000)	>5000	National Tibetan Plateau/ Third Pole Environment Data Center. https://data.tpdc.ac.cn/en/data/f2aab6ba-9b57-4d77-b64e-8422282316a8	1km
Slope (°)	≥25	(15, 25)	(8, 15)	≤8		1km
Freeze-Thaw Erosion	Freeze-Thaw Erosion Zone		Non-Freeze-Thaw Erosion Zone		http://data.tpdc.ac.cn [43]	1km
Precipitation (mm)	≥800	(400, 800)	(200, 400)	≤200	https://cstr.cn/18406.11.Meteoro.tpdc.270905 [44]	1km
Vegetation Coverage (%)/NDVI	≥60	(45, 60)	(30, 45)	≤30	https://cstr.cn/18406.11.Ecolo.tpdc.270449 [45]	250m
Land use function	Uncategorized, as the result of remote sensing image interpretation.				https://data.tpdc.ac.cn/en/data/3cdc6d05-e8c1-41e2-94fe-00d679c89f89 [46]	300m

**Fig 2:**
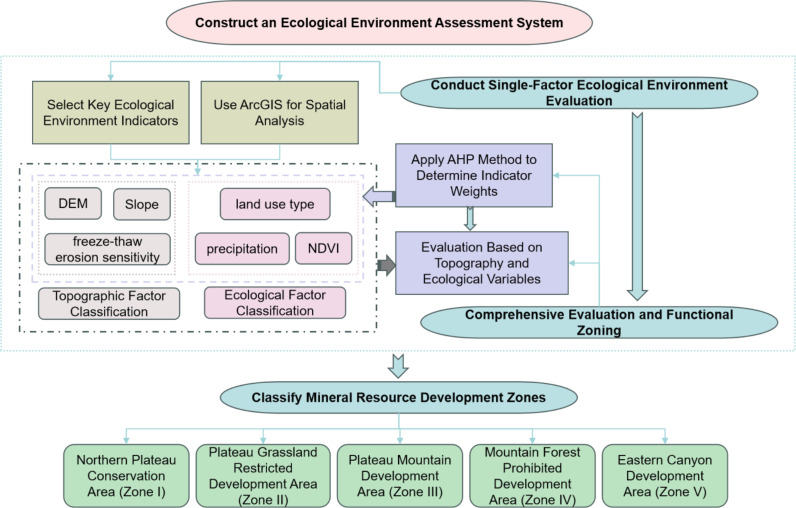
Flowchart of the construction process of the ecological environment assessment system.

This study constructed an ecological environment assessment system to quantitatively evaluate ecological vulnerability (**Fig 2**). Key ecological environment indicators (elevation, slope, freeze-thaw erosion sensitivity, annual average precipitation, land use types, and vegetation coverage) were selected to form the foundational dataset for assessing the ecological environment. The hierarchical structure model was developed based on extensive literature reviews and expert consultations, comprising two primary indicators: topographical factors and ecological factors. The topographical factors included three secondary indicators: elevation, slope, and freeze-thaw erosion; while the ecological factors included three secondary indicators: precipitation, normalized difference vegetation index (NDVI), and land use type. The analytic hierarchy process (AHP) was employed to quantify the relative importance of each indicator and determine their respective weights (**Table 2**). The weights reflect the relative contributions of each indicator to the assessment of ecological vulnerability.

**Table 2:** Ecological environment index and weights.

Indicator System	Weight	Single Indicator Factor	Weight
Topographical Factors	0.35	Elevation (m)	0.35
		Slope (°)	0.3
		Freeze-Thaw Erosion	0.35
Ecological Factors	0.65	Precipitation (mm)	0.25
		Vegetation Coverage (%)	0.4
		Land use function	0.35

During the data collection and preprocessing phase, data on elevation, slope, freeze-thaw erosion, precipitation, NDVI, and land use type were obtained from multiple databases and standardized to eliminate differences in units and ranges among the indicators. Subsequently, each secondary indicator was classified using ArcGIS software. Based on the ecological significance and data distribution characteristics of each indicator, different classification intervals were established for each secondary indicator to more intuitively reflect their contributions to ecological vulnerability.

After the classification process, a hierarchical overlay analysis was conducted using geographic information system (GIS) technology. The standardized data layers of each secondary indicator were weighted and overlaid according to their respective weights to generate a comprehensive map of ecological vulnerability. This map visually presents the spatial distribution of ecological vulnerability across the study area, providing a visual outcome for the assessment of ecological vulnerability. Based on the integrated evaluation results of the ecological environment, in conjunction with the geological conditions of Xizang, ecological information functional zones for mineral resource development were delineated, and the impact of different ecological environments on mineral resource development in Xizang was discussed.

**Fig 3:**
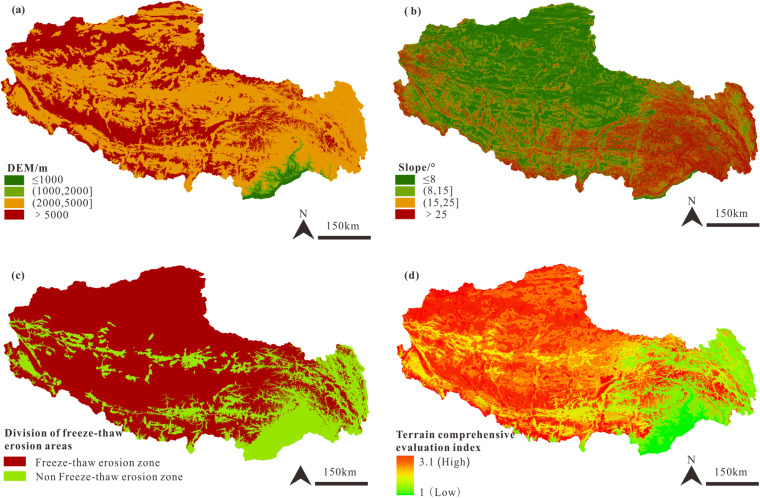
Topographical factor evaluation and discrimination image of Xizang. The images were created with ArcGIS 10.2, URL: http://www.esri.com/software/arcgis/arcgis-fordesktop. (a). DEM regional division. (b). Slope regional division. (c). Division of freeze-thaw erosion areas. (d) Terrain Comprehensive Evaluation Regional Division.

**Fig 4:**
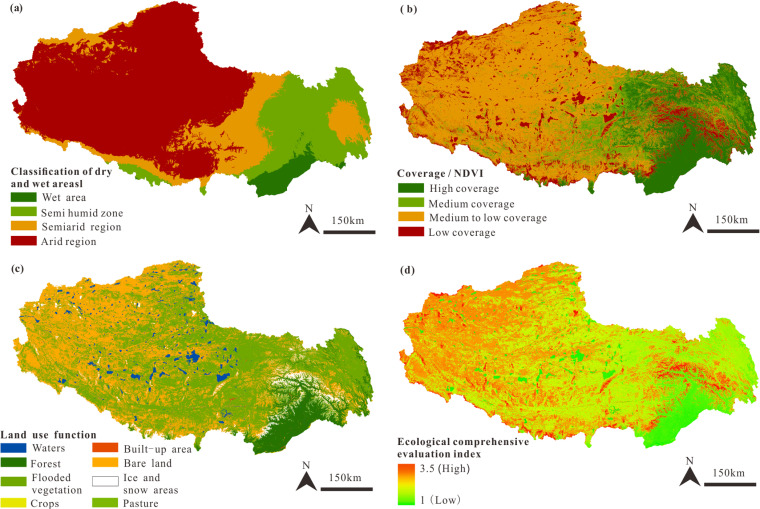
Ecological factor evaluation and discrimination image of Xizang. The images were created with ArcGIS 10.2, URL: http://www.esri.com/software/arcgis/arcgis-fordesktop. (a). Classification of dry and wet areal. (b). Vegetation Coverage Regional Division. (c). Land Use Type Regional Division. (d) Ecological Comprehensive Evaluation Regional Division.

**Fig 5:**
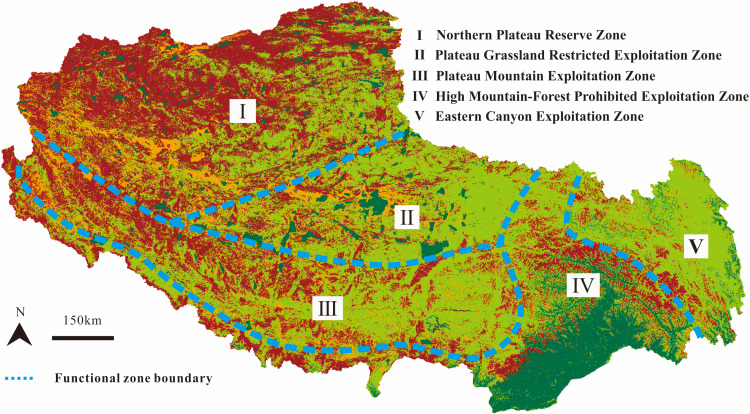
Functional zoning of mineral resource development in Xizang. The image was created with ArcGIS 10.2, URL: http://www.esri.com/software/arcgis/arcgis-fordesktop.

Through this method, we obtained three key output results (Figs 3–5), which display the distribution of ecological vulnerability in different areas. These results provide a scientific basis for further environmental management and decision-making. The following are the grading standards and calculation methods for single-factor indicators.

The harsh climatic conditions of high altitude and low oxygen in Xizang are closely associated with its elevation, which varies significantly across the region. According to China’s geomorphological classification, elevations are categorized into five levels: low altitude (≤1000 m), mid-altitude (1000 m-2000 m), sub-high altitude (2000 m-4000 m), high altitude (4000–6000 m), and extremely high altitude (>6000 m). Based on this classification, and considering that over 95% of Xizang’s area lies above 2000 m, the elevation classification conditions used in this study are listed in **Table 1**.

In areas with steep slopes, the difficulty of mineral resource extraction increases, and soil stability is compromised due to erosion, and thus landslides, debris flows, and other geological disasters are more likely to occur. According to the “Technical Regulations for Land Use Status Survey,” slope classification derived from elevation data divides slopes into gentle slopes (≤8°), moderate slopes (8°-25°), and steep slopes (≥25°). The eastern canyon region of Xizang has steep slopes, whereas the Qiangtang Plateau in the west, although relatively high in elevation, is relatively flat. Based on slope classification standards and the topographical undulations of Xizang and considering the angles required for resource extraction, this study further subdivides moderate slopes, resulting in a four-tier slope classification (**Table 1**).

Freeze-thaw erosion, a significant type of soil erosion in Xizang, disrupts soil structures and increases the risk of landslides, posing safety hazards for infrastructure and transportation routes in erosion-prone areas [47]. Currently, there is no unified grading standard for freeze-thaw erosion. Many scholars have found that calculating the lower boundary of the freeze-thaw erosion zone to be 200 m lower than the permafrost zone is more accurate [48] (Eq. 1).


$$H=\frac{66.3032-0.9197X-0.1438Y+2.5}{0.005596}-200$$ (1)

where H represents the lower boundary elevation of the freeze-thaw erosion zone (m), X is longitude, Y is latitude; H > 0 indicates a freeze-thaw erosion zone, and H < 0 indicates a non-freeze-thaw erosion zone.

Mineral resource development requires abundant water resources; however, increased precipitation can exacerbate soil erosion, damaging infrastructure such as roads. According to China’s humidity classification standards, annual precipitation is categorized into humid areas (≥800 mm), semi-humid areas (400–800 mm), semi-arid areas (200–400 mm), and arid areas (≤200 mm).

The Normalized Difference Vegetation Index (NDVI) is a critical indicator for ecological environment monitoring, reflecting the degree of vegetation cover in different regions. Vegetation cover varies significantly in Xizang, with primary forests in the east, alpine meadows and grasslands in the central region, and high levels of bare ground in the west. Based on the percentage classification standards of vegetation cover by the Food and Agriculture Organization of the United Nations (FAO), the levels are divided into high coverage (≥60%), medium coverage (45%-60%), medium to low coverage (30%-45%), and low coverage (≤30%).

## 3. Results

### 3.1. Ecological environment assessment

#### 3.1.1. Topographical factors.

Amidst the challenging natural conditions including high altitude, low temperatures, arid climate, and oxygen scarcity, Xizang confronts an arduous operational milieu marked by limited accessibility, geographic seclusion, and inadequate infrastructure. These factors collectively result in significant accessibility issues. The widespread distribution of glaciers, steep slopes in the eastern canyons, and poor soil stability increase the risks of collapses or ice avalanches during development, posing significant risks to the safety of on-site personnel. Considering these natural factors, this study evaluates elevation, slope, and freeze-thaw erosion as pivotal topographical variables.

The average elevation of Xizang is about 4000 m, with more than 90% of the region situated above 2000 meters. The terrain generally slopes downward toward the southeast and rises toward the northwest (**Fig 3a**). In southeastern Xizang, due to rugged landforms like the Yarlung Zangbo Grand Canyon, exhibits greater slope steepness (**Fig 3b**). Although the northwestern Xizang has a higher elevation, its terrain is relatively more gently undulating. Glaciers extensively cover Xizang (**Fig 3c**), predominantly concentrated in the northwest and along the southeastern edges, corresponding to areas with higher elevations and steeper slopes, which are more susceptible to such erosion processes.

In alignment with the principles and objectives of comprehensive mineral resource zoning, the study utilizes the AHP in conjunction with expert assessments to assign weights to key topographical indicators, namely the DEM, slope, and freeze-thaw erosion sensitivity, with corresponding weightings of 0.35, 0.3, and 0.35, respectively. This approach results in a synthesized evaluation image that encapsulates Xizang’s topographical factors (**Fig 3d**). The resulting synthesized evaluation image presents the topographical conditions in the form of categorical levels (1–4) rather than continuous numerical values. These levels are derived from the weighted overlay analysis, where each level represents a composite assessment of the terrain based on the combined influence of DEM, slope, and freeze-thaw erosion sensitivity. Specifically, a lower-level number (closer to 1) indicates more favorable topographical conditions, while a higher-level number (closer to 4) suggests less favorable conditions. This categorization facilitates a clearer spatial representation and interpretation of the terrain’s suitability for mineral resource zoning.

The results indicate that the southeastern region of Xizang has relatively favorable topographical conditions. In contrast, the northern area encounters considerable challenges in resource development and utilization, underscoring the need for strategic planning and meticulous integration of topographical factors in resource development and utilization in Xizang.

#### 3.1.2. Ecological factors.

The considerable water usage inherent in the extraction, smelting, and processing stages of mineral resources significantly influences the development of the mineral industry. The precipitation in Xizang is primarily concentrated in the southeastern area, and the western area is characterized by arid conditions (**Fig 4a**). The vegetation coverage in Xizang is generally low, especially in the western areas (**Fig 4b**). Deserts, predominantly composed of exposed rock and bedrock, are primarily distributed in the northern area, where the ecological disturbance from mineral resource development is relatively low. Pasture is predominantly located in central Xizang, including alpine meadows, alpine steppes, and high-cold meadows [23]. Given the delicate ecological balance and the effects of high-altitude hypoxia, vegetation self-restoration is relatively weak, making grassland areas prone to ecological degradation. Forested areas thrive in the eastern canyon areas of the mountain ranges, benefiting from favorable hydrothermal conditions and rich biodiversity (**Fig 4c**). While forest ecosystems are inherently resilient, their role in ecological environment underscores the importance of minimizing human-induced harm during mineral the resource exploitation.

The study employs the AHP and expert evaluation to assign weights of 0.25, 0.4, and 0.35 to precipitation, vegetation coverage, and land use types, respectively, resulting in a comprehensive evaluation image of ecological factors (**Fig 4d**). The values ranging from 1 to 3.5 represent the classification levels derived from the weighted overlay analysis, rather than specific measured values. Lower classification levels (closer to 1) indicate better ecological and environmental conditions, while higher classification levels (closer to 4) indicate poorer ecological and environmental conditions. The overall ecological rating of Xizang is deemed to be relatively low, particularly highlighting the northern area as having the most tenuous ecological conditions.

According to the weight of topographical factors at 0.35 and ecological factors at 0.65, a superimposed analysis of Figs 3d and 4d was conducted to obtain the comprehensive ecological environment evaluation result for Xizang (**Fig 5**). In recent years, Xizang has strengthened its protection of the ecological environment, and large-scale activities (such as mining) have been relatively infrequent, leading to a generally better overall ecological environment in Xizang. The ecological environment of the northern area is more fragile and susceptible to damage than the eastern canyon area. However, due to its unique natural conditions, including climate and altitude, human activities have relatively less impact on the ecological environment in this area. In the context of mineral resource reserves and development, it is essential to carefully evaluate each region’s ecological environment to ensure its sustainability.

### 3.2. Functional zoning

The functional zoning of mineral resource development aims to effectively regulate and guide the exploration and development direction, considering the disparities in regional resources and environmental and economic factors. Such zoning must be flexible and adaptive to local conditions to ensure the sustainable and efficient use of mineral resources. Based on the above results, incorporating key factors such as metallogenic belts, ecological vulnerability, mineral resource distribution, and national development positioning [8,49], this study divides the mineral resource development functional zones in Xizang into distinct zones: Northern Plateau Desert Zone (Zone I), Plateau Grassland Zone (Zone II), Plateau Mountain Zone (Zone III), High Mountain-Forest Zone (Zone IV), and Eastern Canyon Zone (Zone V) (**Fig 5**). Among them, Zone I is designated as an alternative reserve zone for mineral resources. Zone II is recommended to impose temporary restrictions on the exploitation of mineral resources. Zones III and V are deemed relatively suitable for the exploitation and development of mineral resources. Zone IV should prohibit exploration activities to protect the ecological balance.

**Fig 6:**
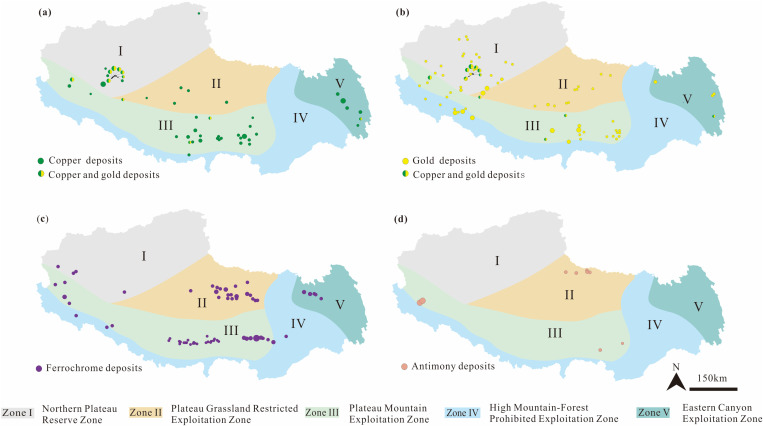
Distribution of resources in the mineral resource development functional areas of Xizang. The image was created with ArcGIS 10.2, URL: http://www.esri.com/software/arcgis/arcgis-fordesktop.

**I. Northern Plateau Desert Zone:** This zone is in the northwestern Xizang and is predominantly characterized by barren lands with extensive areas of exposed rock. The central area predominantly comprises alpine meadows characterized by grasslands featuring sparse vegetation, shallow soil strata, and severe desertification. Glaciers are primarily found in the western mountainous areas, predominantly consisting of permanent ice formations. Additionally, a large area of nature reserves is situated in this zone. Zone I is rich in ferrous metals (such as iron and chromium) and non-ferrous metals (such as copper, lead, and zinc) (**Fig 6**). Lithium deposits are mainly located in the northern lake regions. Typical deposits include the Rongna copper deposit, Zapu chromite deposit, and Mami Cuo lithium borate salt deposit.

Mining activities in Zone I can exacerbate land degradation through soil compaction and topsoil loss. Water scarcity is already a challenge in this zone, and mining operations can further strain limited water resources. To mitigate these impacts, sustainable water management practices, such as water recycling and efficient use technologies, are essential. Revegetation programs should be implemented to restore degraded areas and prevent soil erosion. Dust control measures, like water spraying and vegetation barriers, can help reduce air pollution.

**II. Plateau Grassland Zone:** This zone is in the central north Xizang, and features a relatively gentle topography with sparse vegetation interspersed with numerous salt lakes. This zone primarily comprises alpine meadows and pastures, with significant grassland degradation. Zone II also includes multiple wetland ecosystems, crucial for conserving wildlife [50]. Regarding mineral resources, non-ferrous metal deposits (including copper, lead, and zinc) and noble metal deposits (including gold and silver) are distributed in the central and southern areas. While antimony deposits are concentrated along the north edge of the zone. Typical deposits are the Bolazha copper deposit, Xiongcun copper-gold deposit, and Gaerbakuoer antimony deposit (**Fig 6**).

Mining in Zone II can lead to significant vegetation loss, disrupting grazing lands and wildlife habitats. The removal of vegetation cover increases the risk of soil erosion, especially during heavy rainfall. Moreover, the disruption of grassland ecosystems can lead to a decline in biodiversity. To address these challenges, selective mining techniques should be employed to minimize the area of land impacted. Large-scale grassland restoration projects are necessary to rehabilitate degraded areas. Establishing protected areas and wildlife corridors can help preserve biodiversity.

**III. Plateau Mountain Zone:** This zone is located between the Gangdese Mountains and the northern slope of the Himalayas, with the central region situated on either flank of the Yarlung Zangbo suture zone. This zone is characterized by sparse vegetation, glacier and snow cover in some regions and significant terrain undulations with mostly exposed bedrock. Grasslands, predominantly in the form of alpine meadows, are primarily located in the eastern area. Key urban centers of Xizang, including Lhasa, Shigatse, and Nyingchi, are concentrated in this area, where anthropogenic activities exert a substantial impact on the ecological environment. The area boasts a wealth of non-ferrous metals, including copper, molybdenum, lead, zinc, and ferrous metals such as chromium and iron. Copper-molybdenum deposits predominantly occur in the eastern area, notably the Xiongcun copper deposit and Narusongduo lead deposit (**Fig 6**).

Mining activities in Zone III can trigger geological hazards such as landslides and debris flows, posing risks to infrastructure and human safety. Vegetation disruption from mining can lead to increased soil erosion and habitat loss. Mining runoff can also contaminate local water sources, affecting downstream ecosystems. To mitigate these risks, continuous geotechnical monitoring is necessary to prevent and mitigate landslides. Vegetation restoration programs should be developed to stabilize slopes and restore habitats. Advanced water treatment technologies should be implemented to manage and reduce mining-related water pollution.

**IV. High Mountain-Forest Zone:** This zone is a region of immense ecological importance, including the Himalayas and Tanggula Mountains, characterized by high elevations and perpetual snow cover on the mountain ranges. And the southeastern Xizang primeval forests are also in this zone. The southeastern Xizang primeval forest is located on the southern slope of the Himalayas, receiving abundant precipitation, with high forest coverage and minimal exposed bedrock. This zone is abundant in both biodiversity and water resources. This zone also encompasses nature reserves which are primarily dedicated to the conservation of forest ecosystems. Mineral resources are relatively scarce and scattered in Zone IV. Mining activities are strictly limited to prevent ecological damage.

Even small-scale mining in Zong IV can lead to significant deforestation and habitat fragmentation. Removal of tree cover increases the risk of soil erosion and landslides. Mining activities can introduce pollutants into water sources, affecting downstream ecosystems. To protect this fragile ecosystem, strict no-mining policies should be enforced. Comprehensive ecological monitoring programs should be implemented to assess and mitigate any potential impacts. Sustainable forestry practices should be promoted to maintain ecosystem health and resilience.

**V. Eastern Canyon Zone:** This zone is situated in the easternmost part of Xizang and features a diverse range of geomorphological types. It is characterized by vertical natural zonation that transitions from canyon forests to alpine meadows and snow-capped peaks. The vegetation coverage is relatively high, primarily grasslands, with some areas partially exposed to bedrock. The main mineral resources include copper-molybdenum, chromite, and lead-zinc. Among the significant deposits are the Yulong copper deposit, the chromite deposits in Basu, and the Ganzhongxiong lead-zinc deposit.

Mining activities in Zone V can lead to the contamination of rivers and groundwater, affecting local communities and ecosystems. Steep slopes and heavy rainfall increase the risk of soil erosion, particularly during mining operations. Disruption of forest and meadow ecosystems can lead to the decline of endemic species. To mitigate these impacts, advanced water management systems should be implemented to prevent contamination. Erosion control techniques, such as terracing and vegetation barriers, should be used to stabilize slopes. Protected areas and wildlife corridors should be established to preserve biodiversity and ecosystem health.

## 4. Discussion

The high-altitude areas in Xizang, mostly exceeding 3500 m, poses substantial challenges to human activities due to harsh conditions such as hypoxia, which can lead to altitude sickness and decreased work efficiency. In recent years, climate change, characterized by rising temperatures and increased humidity, has contributed to permafrost degradation, affecting the terrain’s stability and the feasibility of mineral resource development, especially in permafrost regions [51]. The intensification of the hydrological cycle, marked by glacier retreat and increased runoff, can impact mineral resource development, particularly during the rainy season, increasing the difficulty and risk of operations [52]. Taking the Duolong copper deposits in Zone I as an example, the deposit is situated at an altitude of more than 5000 m in the Ali region known as the “forbidden zone of life”. Despite the identified copper resources amounting to 20 million tons [49], these resources have not yet been effectively utilized due to their undeveloped status. The extreme natural environment within the area poses a severe challenge to human survival, leading to a scarcity of labor resources. In addition to the necessity of ecological protection, ecological and environmental factors are the main constraints on developing its mineral resources. In the context of freeze-thaw cycles, the arid desertification ecosystem has sparse surface vegetation and a fragile ecological environment, which is extremely difficult to restore once damaged (Figs 3 and 4). This is precisely why Zone I, despite its abundant mineral resources, is designated as a resource reserve area, whereas Zones III and V are suggested for resource development.

The extreme natural environment of Xizang leads to severe challenges to human survival, especially in the Ali region, where labor resources are scarce. The complex terrain and significant altitude differences lead to transportation difficulties, restricting the movement of people and materials and affecting population aggregation and development [11]. The construction and maintenance of infrastructure under Xizang’s extreme climatic conditions, including low temperatures, strong winds, and reduced precipitation, are challenging. Extreme weather events can lead to transportation disruptions, affecting the supply of materials and the safety of personnel [53]. There may be a lack of necessary technical and human resources to support the development of mineral resources, including professional geological survey personnel, engineers, and technical workers for the operation and maintenance of mining equipment. Most mines globally are located in remote areas with inadequate infrastructure support, and ground communication stations cannot achieve full coverage of mines, leading to weak or no signal, affecting the collection, exploration, and production of mineral resources [54].

Land use types significantly reflect soil erosion intensity. The unutilized lands, including mines and bare lands without vegetation cover, are high-risk areas for soil erosion [55]. Mineral resource development may exacerbate land use changes, affecting soil erosion and the ecological environment. Vegetation coverage significantly impacts soil erosion intensity, surpassing other factors, making areas with low vegetation coverage high-risk zones for soil erosion and limiting mineral resource development activities [56]. Zones II and III have similar topography and ecology, and Zone II is rich in inland lakes. The ecological damage in places such as Nagqu in Zone II due to the previous gold sand deposit mining is relatively severe. After the surface mat layer in Zone II is stripped and disappears, the exposed soil and sandstone are wholly exposed in winter, gradually becoming dust storms in the highland winds, thus losing the function of soil and water conservation and water source conservation, forming the so-called “black soil beach.” Most of the mines in this area have entered the closed pit state and are in the ecological environment restoration period, which will be a long process. Therefore, even with a similar ecological environment, we still consider Zone II a restricted mineral resource development zone.

As an ecological barrier in China, Xizang has strict environmental protection requirements. Mineral resource development must strictly adhere to environmental impact assessments, adding development costs and complexity. The region’s ecological sensitivity demands advanced technology and sustainable practices to minimize environmental impact, particularly in development areas with gentle terrain slopes that reduce geological disaster risks but require careful land management to preserve ecological balance [8].

Mineral resource development often leads to negative impacts such as land occupation, vegetation destruction, and soil erosion, necessitating subsequent ecological restoration. Ecological restoration costs are usually high, especially in regions with complex terrain and harsh climatic conditions like Xizang, where restoration requires significant human, material, and financial resources [56]. The Xizang Grassland Ecological Restoration Governance Pilot Demonstration Project, with an investment of over 8.3 million dollars and a duration of 2 years, covered areas such as Lhasa, Nagqu, and Ali, completing 2000 km^2^ of grassland ecological protection and restoration pilot demonstration area, achieving a vegetation coverage of 60%. However, completing natural succession in cold regions often takes decades or even longer, making vegetation restoration and ecological recovery in Xizang a long-term process that may require several decades to reach a satisfactory state of natural succession [57,58]. Therefore, when conducting mineral resource exploration and development, we must adhere to the principle of prioritizing ecological protection. This study divides the mineral resource development into functional areas based on its ecological environment and considers the cost of ecological restoration. Zone IV has the fewest ore deposits discovered among the five functional areas. Regarding the distribution of mineral belts, there are few large ore deposits in the region. Regarding the ecological environment, Zone IV has a steep terrain slope (**Fig 3b**), distributed with pristine forests (**Fig 4c**), making exploring mineral resources more difficult. The trees in the pristine forests are severely aged, and the ecosystem is highly susceptible to damage. Moreover, the cost of forest ecological restoration is more significant than grassland restoration, and the restoration period is more extended. Therefore, although Region IV has a better comprehensive ecological environment than the other four regions, this paper still designates it as a no-mining zone.

The development of mineral resources in Xizang must be approached with a deep understanding of the ecological constraints and a commitment to sustainable practices. It includes developing adaptive strategies for climate change, integrating sustainable land management practices, adhering to strict environmental protection requirements, and investing in infrastructure and human resources. Only through such a comprehensive approach can we ensure that mineral resource development proceeds in a manner that is both economically viable and ecologically sustainable. Mining in Xizang is a complex issue requiring a comprehensive evaluation of geological, environmental, and economic factors. As an important part of the Tethis-Himalayas metallogenic domain, Xizang is endowed with a wealth of mineral resources, particularly copper, chromium, lead-zinc, and lithium, which are crucial for the nation’s economic development and resource security [3,54].

Xizang’s mineral resources are primarily distributed in Zones I, III, and V, indicating a high potential for development. The suitability of development must also consider the environmental implications. Xizang’s high altitude and fragile ecosystem pose significant challenges [59]. The region’s unique geological and climatic conditions demand advanced technology and sustainable practices to minimize environmental impact [1]. The gentle terrain slopes in development areas, while reducing geological disaster risks, also necessitate careful land management to preserve the ecological balance [8]. The significant rainfall in southeastern Xizang, while beneficial for hydropower and water supply, must be managed to prevent erosion and maintain water quality [60]. Zones III and V are both mineral resource development areas. However, unlike Zone III, Zone V has a more developed water system, and transportation routes often pass through rugged mountain roads. During the mineral resource development process in Zone V, relatively more expenses are incurred in maintaining transportation routes. The southeastern Xizang area receives significant rainfall, providing water resources for hydropower development and mineral exploitation. The diverse types of land use are conducive to ecological protection and the sustainable use of resources. Moreover, the high vegetation coverage in mineral resource development areas helps reduce soil erosion and protect the ecological environment [12].

The development of mineral resources in Xizang is both promising and complex. While the region’s rich mineral endowment offers substantial economic opportunities, the need for sustainable and environmentally conscious practices is paramount. The discussion on suitability must weigh the economic benefits against the environmental costs, ensuring that development proceeds to protect Xizang’s unique and sensitive ecosystem for future generations [1,8,22].

By identifying distinct ecological characteristics and development potentials across five functional zones, this study provides a robust framework for balancing mineral resource exploitation with ecological protection. The zoning approach ensures that development activities are tailored to the specific ecological contexts of different regions, promoting sustainable and balanced growth while minimizing environmental impact. This study also highlights the importance of adopting advanced technologies and sustainable practices to mitigate ecological risks, particularly in zones with high resource potential but significant ecological sensitivity. Furthermore, the findings inform global resource management by presenting a replicable model that integrates comprehensive regional zoning with quantitative ecological assessments. This model can guide the development of sustainable mining practices in other regions facing similar challenges. Additionally, the study underscores the need for strong regulatory frameworks and community engagement to ensure that development benefits are shared equitably and ecological values are preserved. Overall, the functional zoning scheme not only advances the scientific and refined management of resources and the environment in Xizang but also provides valuable lessons for sustainable development in ecologically sensitive areas worldwide.

## 5. Conclusions

This study employs an ecological functional zoning approach to comprehensively analyze the balance between mineral resource development and ecological protection in Xizang. The study divides Xizang into five functional zones, each with distinct ecological characteristics and mineral resource development potential. The Northern Plateau Desert Zone and the Plateau Mountain Zone have relatively favorable conditions for development but face significant ecological vulnerability. The Plateau Grassland Zone and the High Mountain-Forest Zone have sensitive ecosystems that constrain development. The Eastern Canyon Zone has strong ecological restoration capabilities but is susceptible to water contamination and soil erosion risks. The mineral resources in Xizang are crucial for national economic development, but their exploitation must be conducted within the framework of ecological protection. This study provides a scientific basis for sustainable development in Xizang, emphasizing the importance of prioritizing ecological protection and promoting innovation in technology and management. Future efforts should focus on strengthening international cooperation, improving legal frameworks, and enhancing community participation to achieve a win-win situation for the economy and the environment.
